# Efficacy of intraoperative epidural steroids in lumbar discectomy: a systematic review

**DOI:** 10.1186/1471-2474-15-146

**Published:** 2014-05-05

**Authors:** Bakur A Jamjoom, Abdulhakim B Jamjoom

**Affiliations:** 1 Core Surgical Trainee, University Hospital of Coventry and Warwickshire, Walsgrave, Coventry CV2 2DX, UK; 2 Department of Surgery, Section of Neurosurgery, King Khalid National Guards Hospital, P O Box 9515, Jeddah 21423, Saudi Arabia

**Keywords:** Lumbar discectomy, Steroids, Methylprednisolone, Intraoperative, Epidural, Randomized controlled trial, Postoperative outcome

## Abstract

**Background:**

This study is a descriptive review of the literature aimed at examining the efficacy of the use of intraoperative epidural steroids in lumbar disc surgery, a matter that remains controversial.

**Methods:**

The relevant clinical trials were selected from databases and reviewed. The methodological quality of each included study was assessed and graded for perceived risk of bias. All the documented significant and non-significant findings were collected. Our outcome targets were reduction in postoperative pain scores, consumption of analgesia, duration of hospital stay and no increase in complication rates. The variation in the timing of postoperative pain assessments necessitated grouping the outcome into three postoperative stages; early: 0 to 2 weeks, intermediate: more than 2 weeks to 2 months and late: more than 2 months to 1 year.

**Results:**

Sixteen trials that were published from1990 to 2012 were eligible. At least one significant reduction in pain score was reported in nine of the eleven trials that examined pain in the early stage, in four of the seven trials that examined pain in the intermediate stage and in two of the eight trials that examined pain in the late stage. Seven of the nine trials that looked at consumption of postoperative analgesia reported significant reduction while six of the ten trails that examined the duration of hospital stay reported significant reduction. None of the trials reported a significant increase of steroid-related complications.

**Conclusions:**

There is relatively strong evidence that intraoperative epidural steroids are effective in reducing pain in the early stage and reducing consumption of analgesia. There is also relatively strong evidence that they are ineffective in reducing pain in the late stage and in reducing duration of hospital stay. The evidence for their effectiveness in reducing pain in the intermediate stage is considered relatively weak. The heterogeneity between the trials makes it difficult to make undisputed conclusions and it indicates the need for a large multicenter trial with validated outcome measures that are recorded at fixed time intervals.

## Background

Many lumbar discectomy patients experience persistent or recurrent back or leg pain following surgery**.** Epidural steroids have been tried for many years as an adjunct to surgery in lumbar disc disease. Their use under these circumstances has been an attempt to reduce early postoperative inflammatory reaction and late scar formation in order to lessen postoperative pain. Ranguis et al. published a systematic review of 12 trials that examined the subject during 1992–2008 [1]. Four more trials have been published since [2-5]. In addition a survey of 112 Canadian neurosurgeons in 2009 showed that 61% of participants do not use epidural steroids in lumbar discectomy [6]. This would indicate that the clinical use of intraoperative steroids in lumbar discectomy is still a matter of controversy. Hence we feel justified in attempting to provide an updated examination of the literature on the matter.

This study is a descriptive review of the literature directed at examining the efficacy of intraoperative epidural steroids in lumbar discectomy. The objectives are to assess whether the use of steroids under such circumstances has significant effect on the severity of postoperative pain, the extent of analgesia consumption, the duration of hospital stay and the complication rates. We aim to achieve these goals by identifying and grouping all the significant differences relating to the outcome targets between patients who had intraoperative epidural steroids and controls as described by the authors.

## Methods

### Eligibility criteria

Our inclusion criteria were randomized controlled trials or cohort studies of patients who underwent lumbar discectomy and had steroids applied onto the epidural and exposed nerve root intraoperatively. The review was limited to studies that were published in the English language up to 2012. We included studies that provided sufficient data relating to all or part of the following: assessment scores for back pain (BP) and radicular leg pain (RLP) at defined times in the postoperative period, records of the extent of postoperative analgesia usage, the duration of hospital stay and complication rates. Suitable studies were included irrespective of whether the patients also received steroids intravenously, the discectomy techniques, the steroids dosage, the addition of another medication with the steroids and the inclusion in the trial of another group that received a non-steroidal medication. However, we excluded studies that were not published as full articles [7], studies that were not published in English [8], studies that reported patients in whom the steroids were injected intramuscularly (IM) [9] or intravenously (IV) [10,11] without an epidural application. We also excluded studies of lumbar disc patients that had epidural steroids without surgery [12] and those in which the patients were treated with non-steroidal medications whether epidural [13,14], IV [15] or IM [16].

### Literature search

The literature was systematically searched in April 2013 using a combination or part combination of the following terms: intraoperative, perioperative, epidural, steroids, methylprednisolone, depomedrol, lumbar disc surgery, discectomy for herniated lumbar disc, postoperative back pain and radicular leg pain and randomized controlled trials. The two investigators independently interrogated the literature using the databases PubMed, Medline and the Cochrane Central Register of Controlled Trials. The full texts of the potentially appropriate studies were retrieved and assessed.

### Data extraction

Data were extracted from the included studies using a standardized form. The two investigators performed this independently and compared results to reduce extraction error. Missing data were referred to as not available. The following data were collected for each study: year of publication, number of patients treated with steroids, number of controls, total number of patients in the trial, dose of steroids and any additional medications given, method of pain score assessment, all recorded postoperative pain scores for BP and RLP and their timing, record of consumption of postoperative analgesia, duration of hospital stay and rates of complications such as infection and disc prolapse recurrence.

### Outcome measures

Our outcome targets were: reduction in the postoperative pain scores for BP and RLP, reduction in the postoperative consumption of analgesia, reduction in the duration of hospital stay and no increase in complication rates.

### Data analysis

As a result of the variation in the methods of pain scoring and timing assessments between the various series, the analysis was descriptive and focused on collecting all the significant and non-significant differences between the steroids and control groups as documented by the authors. The variation in the timing necessitated grouping the pain score assessments them into three postoperative stages: Early: from 0 to 2 weeks, Intermediate: from more than 2 week to 2 months and Late: from more than 2 months to 1 year after surgery. The evidence for or against each outcome target was considered strong or weak based on the number of supportive trials in comparison to the non-supportive trials, the size of their total patient population and their year of publication.

### Assessment of methodological quality

The two authors independently assessed the methodological quality of the reviewed articles based on a number of criteria including: randomization, blinding, withdrawals and dropouts, description of exposed and unexposed patient characteristics, comparability of cohorts, inclusion and exclusion criteria and definition and objectivity of outcomes. Each study was subjectively scored 0 or 1 for each of the mentioned criteria. Based on the score and on discussion between the two investigators if the scores varied, each study was graded for its perceived risk of bias as low risk, moderate risk or high risk.

## Results

### Study selection and characteristics

The literature search yielded 16 studies of lumbar discectomy and intraoperative application of epidural steroids that were considered suitable for review. These included a total of 693 patients that had lumbar discectomy and received intraoperative epidural steroids and 617 controls [2-5,17-28]. The steroids used were methylprednisolone 40 mg [3,4,17,18,21,24,26], methylprednisolone 80 mg [2,5,19,20,22,23,25,27] and dexamethasone 16 mg [28]. The additional medications used included epidural fentanyl [5] and morphine [2,23,24] as well as IM bupivacaine [21,25] and IM and IV methylprednisolone [20,25]. The postoperative pain scores were assessed by Visual Analog Scale (VAS) [3-5,18,20-22], by McGill Pain Questionnaire (MPQ) [2,23], by Aberdeen Back Pain Index (ABPI) [2] or by using a Numerical Rating Scale (NRS) from 0 to 10 [17,19], from 0 to 3 [26] and from I to V [25]. Some authors did not state their methods of grading pain [24,28].

### Results of individual studies and risk of bias

Table 1 summarizes all the significant and non-significant pain scores for treated patients and controls at the specified postoperative times as documented by the authors and the bias risk grade for each series. Analysis of the series reporting significant and non-significant reduction in pain scores during the early, intermediate and late postoperative stages is summarized in Table 2. Analysis of the series reporting significant and non-significant reduction in the consumption of postoperative analgesia and in the hospital stay is summarized in Table 3. None of trials reported a significant steroids-related increased rate of infection or recurrences. There were few reports of complications such as superficial wound infection, erythema, serous discharge, reoperation for recurrence and readmission for pain management that were observed nearly equally in both groups [2,17,20,23].

**Table 1:** Analysis of the significant and non-significant pain score recordings in the various series

**Authors (Year) [Reference]**	**Patients numbers steroids/control**	**Bias risk grade**	**Significant pain score reduction**	**No significant pain score reduction**
Diaz et al. (2012) [2]	99/52*	Low	BP/RLP 1D,3D,7D	BP/RLP 3 W, 6 W, 12 W, 6 M, 12 M
Abrishamkar et al. (2011) [3]	22/22*	Moderate	BP 6-12H and RLP 6H	BP 18H, 24H, 2 W and RLP12H,18H, 24H, 2 W
Modi et al. (2009) [4]	29/28	Moderate	BP 2 W, 1 M	BP 3 M, 6 M, 1Y
Hackel et al. (2009) [5]	85/82	Moderate	BP/RLP 24H, 2-3D	BP/RLP 4-5D, 12 M
Rasmussen et al. (2008) [17]	100/100	Low	RLP 2 M, 1Y	BP 2 M, 1Y
Lotfinia et al. (2007) [18]	50/50*	Low	Not available	BP and RLP 24H, 48H, 72H, 96H
Jirarattanaphochai et al. (2007) [19]	17/17	Low	RLP 24H, 48H, 72H, 1 W	BP 1 W, 1 M, 3 M and RLP 1 M, 3 M
Lundin et al. (2003) [20]	38/42	Moderate	BP/RLP 2 W, 6 W, 12 W, 26 W, 52 W**	BP/RLP 2 W, 6 W, 12 W, 26 W, 52 W***
Mirzai et al. (2002) [21]	22/22	Low	Not available	BP 1H, 3H, 6H, 12H
Debi et al. (2002) [22]	26/35	High	BP 1D, 2D, 6D,14D	BP 1Y and RLP 1D, 2D, 6D, 14D
Hurlbert et al. (1999) [23]	30/30	Low	BP/RLP 1D, 3 W, 6 W	BP/RLP 12 W
McNeill et al. (1995) [24]	56/25*	Moderate	Not available	Not available
Glasser et al. (1993) [25]	12/10*	High	BP 1D and RLP 1D	BP 1 W, 1 M and RLP 1 W, 1 M
Lavyne et al. (1992) [26]	42/36	Moderate	Not available	Not available
Davis et al. (1990) [27]	43/43	High	Not available	Not available
Foulkes et al. (1990) [28]	22/23	High	Not available	Not available

*Abbreviations*, *BP* Back pain, *RLP* Radicular leg pain, *H* Hour, *D* Day, *W* Week, *M* Month, *Y* Year.

*Includes other group(s). **Significant worst pain last week. ***Not Significant Pain just now.

**Table 2:** Analysis of series reporting significant and non-significant reduction in the postoperative pain score

**Series reporting**	**Early outcome**			**Intermediate outcome**			**Late outcome**		
	**(1Hour-2Weeks)**			**(+2Weeks-2Months)**			**(+2Months-1Year)**		
	**Total series number [References]**	**Total patients number**	**Median publication year**	**Total series number [References]**	**Total patients number**	**Median publication year**	**Total series number [References]**	**Total patients number**	**Median publication year**
Significant reduction in pain score	**9**	758	2007	**4**	397	2006	**2**	280	2006
	[2-5,19,20,22,23,25]			[4,17,20,23]			[17,20]		
Non-significant reduction in pain score	**2**	194	2005	**3**	267	2007	**6**	580	2008
	[18,21]			[2,19,25]			[2,4,5,19,22,23]		
No pain score	**5**	519	1992	**9**	807	2002	**8**	611	1994
	[17,24,26-28]			[3,5,18,21,22,24,26-28]			[3,18,21,24-28]		

**Table 3:** Analysis of series regarding reduction in postoperative analgesia and hospital stay

**Authors (Year) [Reference]**	**Total patients**	**Reduction in postoperative analgesia**	**Reduction in hospital stay (average in days)**
Diaz et al. (2012) [2]	201	Significant	Not significant
Abrishamkr et al. (2011) [3]	66	Not available	Not available
Modi et al. (2009) [4]	57	Not available	Not available
Hackel et al. (2009) [5]	167	Not available	Significant (4.5 vs. 5.2)
Rasmussen et al. (2008) [17]	200	Not available	Significant (6 vs. 8 )
Lotfinia et al. (2007) [18]	150	Not available	Not available
Jirarattanaphchai et al. (2007) [19]	34	Significant	Not available
Lundin et al. (2003) [20]	80	Not available	Significant (1.7 vs. 2.3)
Mirzai et al. (2002) [21]	44	Significant	Not available
Debi et al. (2002) [22]	61	Not available	Not significant
Hurlbert et al. (1999) [23]	60	Significant	Not significant
McNeill et al. (1995) [24]	110	Not significant	Not available
Glasser et al. (1993) [25]	32	Significant	Significant (1.4 vs. 4)
Lavyne et al. (1992) [26]	78	Not significant	Not significant
Davis et al. (1990) [27]	86	Significant	Significant (2.7 vs. 4.4)
Foulkes et al. (1990) [28]	45	Significant	Significant (6.4 vs. 8.7)

## Discussion

Good pain control following surgery for degenerative lumbar disease is important as it is associated with a decrease in the incidence of postoperative complications [1]. Pain following disc surgery is related to a number of factors that include the inflammatory cascade which is triggered by tissue trauma and direct manipulation of the nerve root. Intraoperative epidural steroids have been used as an adjuvant pain therapy in lumbar disc surgery. It is thought that they reduce postoperative pain by suppressing mediators of pain and inflammation such as prostaglandins, leukotrienes, bradykinin and histamine [2,28]. It is also hypothesized that steroids decrease pain by preventing of epidural fibrosis and limiting the degree of scar formation after lumbar surgery [2]. The latter suggestion however was not supported by the study of Hackel et al. who reported that the application of epidural steroids was not associated with lower incidence of scar formation or failed back syndrome [5].

Intraoperative epidural steroids have been advocated for more than two decades [27,28] and despite the publication of a number of trials their use is still considered a matter of debate. Ranguis et al. published the first systematic review of 12 trials on the topic in 2010. However, their meta-analysis of data related to back pain was limited to 7 trials, data related to radicular pain was limited to 5 trials, data related to postoperative consumption of analgesia was limited to 7 trials and data related to length of hospital stay was limited to 4 trials [1]. This study aimed at assessing the collective experience of all the 16 relevant trials and because of the heterogeneity of the reported data the review was descriptive.

Our review shows that the trials which did not report pain scores compared to those which reported significant and non-significant recordings at the various postoperative stages were relatively older (median publication 1992 vs. 2005, 2007 for early outcome, 2002 vs. 2006, 2007 for intermediate outcome and 1994 vs. 2006, 2008 for late outcome) (Table 2).

In the trials that examined the early outcome for pain scores (Table 2), a significant reduction was observed in 9 out of 11 trials (82%), that had a total patient population of 758 out of 952 (80%) and were relatively more recent compared to those reporting a non-significant reduction (median publication 2007 vs. 2005). This would indicate that the evidence in support of intraoperative epidural steroids reducing early postoperative pain (within the first two weeks) can be considered relatively strong.

In the trials that examined intermediate pain score outcome (Table 2), a significant reduction was observed in 4 out of 7 trials (57%), that had a total patient population of 397 out of 664 (60%) and were of comparable publication year to those reporting a non-significant reduction (median publication 2006 vs. 2007). This would indicate that the evidence in support of intraoperative epidural steroids reducing intermediate postoperative pain (from two weeks to two months) can be considered relatively weak.

In the trials that examined late pain score outcome (Table 2), a non-significant reduction was observed in 6 out of 8 trials (75%), that had a total patient population 580 out of 860 (67%) and were relatively more recent compared to those reporting a significant reduction (median publication 2008 vs. 2006). This would indicate that the evidence in support of intraoperative epidural steroids not reducing late postoperative pain (from 2 months to one year) can be considered relatively strong.

In the trials that examined the consumption of postoperative analgesia (Table 3), a significant reduction was observed in 7 out of 9 trials (77%) that had a total patient population of 502 out of 690 (73%) and were relatively more recent compared to those reporting a non-significant reduction (median publication 1999 vs. 1994). This would indicate that the evidence for intraoperative epidural steroids reducing the consumption of postoperative analgesia can be considered relatively strong.

In the trials that examined the duration of hospital stay (Table 3), a significant reduction was observed in 6 out of 10 trials (60%) that had a total patient population of 610 out of 1010 (60%) and were relatively older than those reporting a non-significant reduction (median publication 1998 vs. 2001). This would indicate that the evidence for intraoperative epidural steroids reducing the duration of hospital stay can be considered relatively weak.

Our review also endorses that the use of intraoperative steroids is not associated with an increased risk of complications such as infection and prolapse recurrence. Lowell et al. reported three cases of epidural abscess that occurred following the use of epidural steroids in 31 micro discectomy patients [29]. They suggested that the infection may have been related to the use of epidural steroids. Their findings however have not been substantiated by others reporters or by our review.

Our results support the conclusions made by Ranguis et al. that intraoperative epidural steroids decrease pain in the short term and reduce the postoperative consumption of analgesia [1]. However it did not support their observation that steroids use is associated with a significant shortening of hospital stay. The latter could be because they based their conclusion on the data of 4 trials only [1].

### Study limitations

There are several important limitations in the available literature on intraoperative epidural steroids in lumbar disc surgery. This is mainly because the various studies were heterogeneous with regards to the outcome measures, the method of pain assessment, the timing and location of the pain assessed, the surgical technique, the steroid dosage, the addition of other medication, the reporting of all relevant data and the risk of bias. As a result we elected not to address the matter of timing of return to work and quality of life in this study.

## Conclusions

The considerable variation between the trials makes it difficult to make undisputed conclusions. Nevertheless, based on the assessment of 16 intraoperative epidural steroids trials it appears that there is relatively strong evidence that they are effective in reducing pain in the early stage and reducing the consumption of postoperative analgesia without an increased risk of complications. There is also relatively strong evidence that they are ineffective in reducing pain in the late stage and in reducing the duration of hospital stay. The evidence for their effectiveness in reducing pain in the intermediate stage is considered relatively weak. Our findings support the use of epidural steroids in lumbar discectomy. However, there is a definite need for a large multicenter trial with validated outcome measures that are recorded at fixed time intervals.

## Abbreviations

BP: Back pain; RLP: Radicular leg pain; IM: Intramuscularly; IV: Intravenously; VAS: Visual analog scale; MPQ: McGill pain questionnaire; ABPI: Aberdeen back pain index; NRS: Numerical rating scale; H: Hour; D: Day; W: Week; M: Month; Y: Year.

## Competing interests

The authors declare that they have no competing interests.

## Authors’ contributions

BAJ: Data acquisition, analysis and writing of manuscript. ABJ: Study conception, design, data acquisition, analysis and manuscript revision and approval. Both authors read and approved the final manuscript.

## Pre-publication history

The pre-publication history for this paper can be accessed here:

http://www.biomedcentral.com/1471-2474/15/146/prepub
